# Convergent Loss of Prothoracicotropic Hormone, A Canonical Regulator of Development, in Social Bee Evolution

**DOI:** 10.3389/fphys.2022.831928

**Published:** 2022-02-15

**Authors:** Claudinéia P. Costa, Naoki Okamoto, Michael Orr, Naoki Yamanaka, S. Hollis Woodard

**Affiliations:** ^1^Department of Entomology, University of California, Riverside, Riverside, CA, United States; ^2^Life Science Center for Survival Dynamics, Tsukuba Advanced Research Alliance, University of Tsukuba, Tsukuba, Japan; ^3^Key Laboratory of Zoological Systematics and Evolution, Institute of Zoology, Chinese Academy of Sciences, Beijing, China

**Keywords:** bees, development, evolution, hormones, sociality

## Abstract

The evolution of insect sociality has repeatedly involved changes in developmental events and their timing. Here, we propose the hypothesis that loss of a canonical regulator of moulting and metamorphosis, prothoracicotropic hormone (PTTH), and its receptor, Torso, is associated with the evolution of sociality in bees. Specifically, we posit that the increasing importance of social influences on early developmental timing in social bees has led to their decreased reliance on PTTH, which connects developmental timing with abiotic cues in solitary insects. At present, the evidence to support this hypothesis includes the absence of genes encoding PTTH and Torso from all fully-sequenced social bee genomes and its presence in all available genomes of solitary bees. Based on the bee phylogeny, the most parsimonious reconstruction of evolutionary events is that this hormone and its receptor have been lost multiple times, across independently social bee lineages. These gene losses shed light on possible molecular and cellular mechanisms that are associated with the evolution of social behavior in bees. We outline the available evidence for our hypothesis, and then contextualize it in light of what is known about developmental cues in social and solitary bees, and the multiple precedences of major developmental changes in social insects.

## Introduction

Complex social lifestyles have evolved a limited number of times in animals, including more than 17 times in arthropods (Crozier and Pamilo, 1996). Within the insects, complex sociality has evolved most frequently in the bees (Anthophila, Order Hymenoptera). It is estimated that four or more independent bee lineages (within the subfamilies Apinae and Xylocopinae in the family Apidae, and in the tribes Halictini and Augochlorini in the subfamily Halictinae, family Halictidae) have transitioned to the most complex form of sociality, termed eusociality (Batra, 1966; Michener, 1969). This classification refers to species with a reproductive division of labor (i.e., queen and sterile worker castes), overlapping generations of females in the nest, and cooperative brood care among adult females (Michener, 1969). In addition to the eusocial lineages, a variety of additional levels of sociality can be observed in the extant bee taxa, including solitary, communal, quasisocial, and semisocial species, among other social forms (Michener, 1974). Unraveling how sociality evolves in bees and other insects, including its origins and elaborations, remains a major topic of study in biological research (e.g., Cardinal and Danforth, 2011; Berens et al., 2015; Toth and Rehan, 2017).

Studies on eusocial insect evolution have revealed a number of fundamental, and sometimes surprising, features of how sociality evolves. One insight gained is that previously-existing genetic architecture that controls traits in solitary insects is often co-opted in the evolution of novel social phenotypes (Toth and Robinson, 2007; Kapheim, 2016). This co-option can occur, for example, through modifications in the non-coding regulatory regions controlling gene expression (Kapheim et al., 2015; Søvik et al., 2015), changes in protein coding sequences (Hunt et al., 2010; Woodard et al., 2011), or through persistent changes in epigenetic regulation (Yan et al., 2014; Oldroyd and Yagound, 2021). Sometimes, these evolutionary changes involve modifications in core processes whose functions might be predicted to be more conserved, given their fundamental roles in organismal processes. For example, juvenile hormone (JH), which controls reproductive maturation in insects, has lost this gonadotropic function in the highly eusocial honey bee and has taken on new social roles in this bee lineage, such as mediating behavioral development in workers, which includes the age-related transition from working inside to outside the nest (Robinson, 1987; Robinson and Vargo, 1997). An alternative framework in social insect molecular research invokes the role of novel genes, which can originate through processes such as gene duplication and exon shuffling (Long, 2001; Long et al., 2003). Novel genes associated with social evolution have been identified in many lineages where high quality genome sequences are available, and although their functions are typically currently unknown, there is evidence that they might be particularly likely to play functions in sociality. For example, in honey bees, novel genes are strongly expressed in tissues that are either novel or are modified to serve social functions, such as the sting and Nasonov glands (Johnson and Tsutsui, 2011; Jasper et al., 2015). Further support for the significance of novel genes in insect social evolution comes from Shell et al. (2021), who found that the evolution of sociality in bees is strongly associated with gene family expansion within social bee genomes.

Gene loss is a third, less well-understood phenomenon that might be important in the evolution of eusocial lineages. Complex eusociality has famously been hypothesized to be a “point of no return,” or stage in an evolutionary trajectory at which lineages, once fully and obligately eusocial, are unable to return to a less complex, non-eusocial lifestyle (Wilson and Hölldobler, 2005). A genomic prediction of this hypothesis might be the loss of genes that hold important functions in the context of solitary lifestyles, occurring in concert with the transition to complex eusociality. Gene loss under these scenarios might be driven by relaxed constraint and drift or even by natural selection (the “Black Queen Hypothesis”; Morris et al., 2012; Albalat and Cañestro, 2016). Based on comparative genomic analyses in insects, gene loss appears to be a pervasive phenomenon, including between relatively closely related species (e.g., among drosophilid species; Hahn et al., 2007). The historical lack of focus on gene loss in social insect evolution is perhaps not surprising, given that it is more intuitive to consider that novel genetic changes would beget novel phenotypes. Additionally, gene loss can have dramatic, widespread, and often harmful effects on organismal function, as is evidenced by comparative functional studies (Carroll, 2008; Roscito et al., 2018). Further, as described above, many examples of genomic, and corresponding phenotypic, novelty have been identified in comparative research. Lastly, gene loss can only be validated with high quality genomic data, where there is a high level of confidence that a gene is truly no longer present in a genome, rather than difficult to detect based on limited data. Despite the caveats, gene loss may also play an important, albeit under-studied, role in eusocial evolution.

## Hypothesis and Supporting Evidence

**Figure 1:**
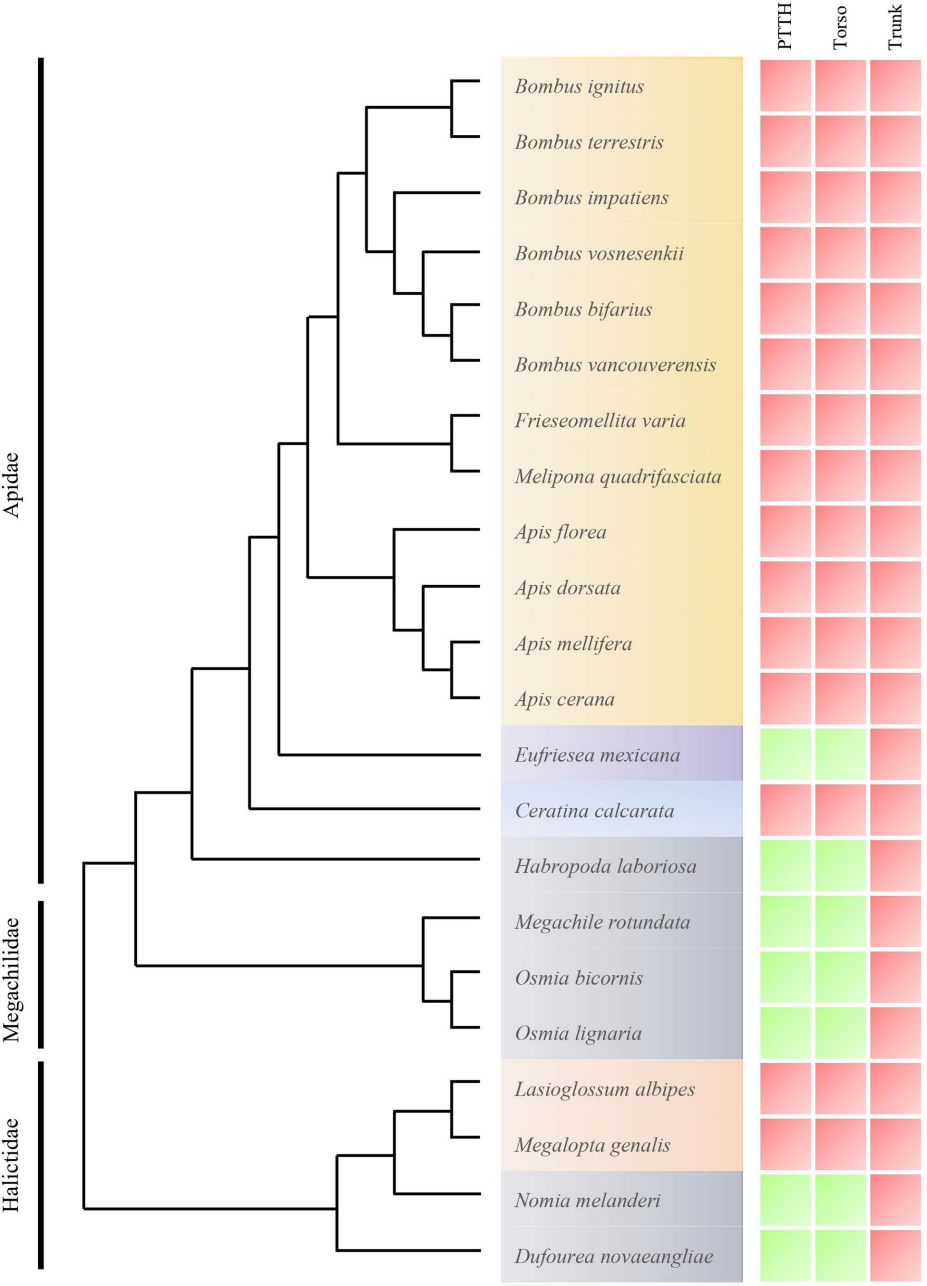
Phylogeny of bees selected for presence and absence of Torso-activation cassette components analysis in bee genomes. We have marked each gene as present (green) or absent (red) in each species. Species names are colored according to degree of social complexity: gray: solitary (ancestrally, and also now); blue: subsocial; purple: communal; orange: facultative eusocial; and yellow: eusocial. *Eufriesea mexicana* is communal, although the details of its social biology are relatively unknown (Cardinal and Danforth, 2011). Phylogenetic relationships between social and solitary bees were based on the best current phylogenetic data available for the Hymenoptera (Cardinal et al., 2010; Kapheim et al., 2015; Branstetter et al., 2017).

Here, we report a striking pattern of loss of an important developmental gene detected in eusocial bee genomes. Specifically, we found that a fundamental regulator of moulting and metamorphosis, the neuropeptide prothoracicotropic hormone (PTTH), and also its receptor Torso, have been lost from a number of bee lineages (an estimated 15 species within two bee families, Apidae and Halictidae, for which genomes are available; Figure 1). PTTH is one of the three major hormones described in the classical scheme of insect endocrinology (Doane, 1973), whose primary function is to promote biosynthesis of the moulting hormone ecdysone in the prothoracic glands (Yamanaka, 2021). PTTH released from brain neurosecretory cells binds to Torso on the surface of prothoracic gland cells. Torso is a receptor tyrosine kinase, which phosphorylates downstream kinases, such as Ras, Raf, and ERK to induce expression of downstream target genes including those that encode ecdysone biosynthetic enzymes (Rewitz et al., 2009). The activity of the PTTH-producing brain neurosecretory cells is controlled by abiotic cues such as photoperiod, as evidenced by their association with pacemaker neurons (McBrayer et al., 2007; Vafopoulou et al., 2007; Selcho et al., 2017). PTTH thus regulates ecdysone production and thereby controls developmental timing in response to environmental signals, which is best represented by its regulatory role in pupal diapause in some lepidopteran species (Mizoguchi et al., 2013; Yamada et al., 2016). It has also been shown that PTTH controls larval light avoidance behavior during the wandering stage before pupation in flies and beetles (Yamanaka et al., 2013; Meng et al., 2019), further demonstrating its role as a mediator between the environment and developmental transitions during insect growth. Developmental delay observed in *ptth* mutant insects (Uchibori-Asano et al., 2017; Shimell et al., 2018) suggests that PTTH is critical for upregulating ecdysone production and maintaining normal developmental schedule in accordance with environmental conditions, but it is dispensable for ecdysone production *per se*. Given these known roles of PTTH, it can be described as allowing organisms to flexibly respond to abiotic conditions and time their life history transitions in accordance with environmental cues, such as the onset of winter, that are important for their survival (Shimell et al., 2018).

Skelly et al. (2019) first noted the loss of PTTH from several Hymenopteran genomes, where it was described that multiple single or combined losses of PTTH, and also Torso and Trunk, have occurred in diverse arthropod lineages. These losses are often correlated in that both ligands and receptors are missing from genomes of the same species. To further explore this pattern, we analyzed currently publicly available, high quality bee genomes (*n* = 22) to examine patterns of presence or absence of the genes encoding PTTH, its receptor Torso, and another ligand for Torso named Trunk that is involved in embryonic patterning in some insects (Duncan et al., 2013). Here, with the exception of the *Frieseomelitta varia* genome, which we obtained from Genbank (accession number PRJNA528016), we accessed bee genomes from the Hymenoptera Genome Database (http://HymenopteraGenome.org; Walsh et al., 2021), a genomic database of fully sequenced and annotated hymenopteran insect species that allows for detailed examinations of the presence or absence of genes and pathways during social evolution. We excluded other publicly available bee genomes from our analysis due to the incomplete nature of some of these genomes; it is difficult to differentiate between true gene loss or assembly error. Our examination used the methods of Skelly et al. (2019), but built on this analysis by including an additional 12 bee genomes. We ran _T_BLAST_N_ (Altschul et al., 1990) using either partial or complete peptide sequences from *Drosophila melanogaster*, *Nasonia vitripennis*, and *Megachile rotundata* (the bee species for which *ptth* and *torso* were already identified in Skelly et al., 2019). This was performed for each gene from TAC (Supplementary Table S1) to identify orthologues that were not annotated as peptide sequences in all fully-sequenced social bee genomes and all available genomes of solitary bees (Supplementary Table S2). The corresponding peptide sequence was BLAST searched with an *E*-value cut-off of 0.00001 using the National Center for Biotechnology Information (NCBI) non-redundant database limited to arthropods. Following the procedure used by Skelly et al. (2019), if nucleotide sequence databases of species were not identified, then the Augustus web interface (Stanke and Morgenstern, 2005) was used to create *de novo* models from scaffolds identified in the BLAST search as containing a gene sequence. Augustus creates *de novo* gene models for missing or partial models based on the gene prediction model of the most closely related species available. Parameters used in Augustus include: both strands, partial gene model, and single stranded predictions. Predicted models were BLASTed against the NCBI non-redundant database limited to arthropods to assess similarity to genes of interest in all fully-sequenced social bee genomes and all available genomes of solitary bees, using an *E*-value as cut-off of 0.00001. PTTH amino acid sequences were imported to the MEGA program version 11 (Stecher et al., 2020; Tamura et al., 2021) for building multiple sequence alignments (MUSCLE). Phylogenetic relationships between social and solitary bees were based on the best current phylogenetic data available for the Hymenoptera (Cardinal et al., 2010; Kapheim et al., 2015; Branstetter et al., 2017). Our use of these methods revealed that both PTTH and its receptor are missing from the genomes of all social bees, with the exception of *Eufriesea mexicana*, whose *ptth* gene is present, corroborating previous findings (Skelly et al., 2019). The social biology of *E. mexicana* has been less thoroughly studied than other species in our analysis, but can be best described as weakly social, given that it is not solitary, but also not eusocial, as is true of many orchid bee species (Cameron, 2004).

When considering phylogenetic relationships between the bee species analyzed, and also their level or form of sociality, multiple patterns emerge. First, PTTH and Torso appear to have been lost multiple times in bees, including a minimum of three times in the bee lineages included in our study. These losses occurred in the following lineages: (1) once in the halictid bee lineage leading to *Lassioglosum albipes* and *Megalopta genalis*; (2) another in the apid lineage leading to *Ceratina calcarata*; and (3) once in the Bombini + Meliponini + Apini clade of the corbiculate bees. These three losses represent the most parsimonious evolutionary scenario given the phylogenetic relationships between bee families based on Peters et al. (2017) and Branstetter et al. (2017); the Apidae phylogeny based on Romiguier et al., 2016 and Bossert et al. (2019); and the much greater likelihood of gene loss vs. gain. In contrast, we failed to detect Trunk in any bee lineage included in this study, further supporting the close association between the losses of Torso and PTTH.

A second pattern detected within our analysis is that within bees, the loss of PTTH and Torso appears to have occurred in lineages that are either eusocial (Bombini + Meliponini + Apini, and *Lasioglosum albipes* and *Megalopta genalis*; Michener, 2000) or subsocial (*Ceratina calcarata*; Rehan and Richards, 2010), whereas all solitary species have retained PTTH and Torso, as has the weakly social orchid bee *Eufriesea mexicana*. Of these four lifestyle classifications, eusociality is the most complex form of sociality, whereas subsociality is typified as exhibiting extended parental care. In the solitary lifestyle, social interactions may occur but largely in the context of mating or other, less sustained interactions.

Based on our finding that the loss of PTTH and Torso has occurred in multiple lineages with more complex sociality (subsocial and eusocial lineages), we propose an evolutionary explanation for this pattern, in light of bee social biology. Specifically, we hypothesize that in social bees, an increased reliance on social cues for the regulation of early development led to relaxed constraint on PTTH, followed by the loss of this gene and its functional role. Our hypothesis is based on the following lines of evidence, in addition to the patterns of loss of PTTH and Torso that we detected. Most eusocial bees live in cavities or other spaces where abiotic cues, such as light, are relatively limited, and where other conditions, such humidity and temperature, are tightly regulated by social group members (Danks, 2002; Stabentheiner et al., 2021). Temperature in particular is highly controlled in eusocial bee nests, where thermal conditions of developing brood are maintained by close contact with incubating adults (Heinrich, 1972; Engels et al., 1995; Bujok et al., 2002). Thus, in social bees, socially-regulated conditions within the nest help buffer developing brood against some of the abiotic cues that might otherwise interact with the PTTH and Torso system. Rather than being heavily influenced by abiotic cues, developing brood in social bee nests is instead strongly influenced by social behaviors within the nest. For example, in bumble bees, adults in the nest generate heat using the thorax and transfer this heat to brood through a characteristic incubation behavior that influences brood development rates (Heinrich, 1972, 1975). Additionally, patterns of larval feeding by brood-rearing adults in bumble bee nests influence the timing of larval development and pupation (Plowright and Pendrel, 1977), body size determination in workers (reviewed in Chole et al., 2019), and the differentiation into the queen and worker castes (Pereboom et al., 2003), which have dramatically different developmental patterns. Thus, both temperature and nutritional conditions for larvae are largely mediated by adults in the nest, rather than more directly reflecting environmental conditions outside of the nest. Moreover, whereas the overwhelming majority of bees are solitary and undergo diapause during early developmental stages, social bee lineages are far more likely to have lost diapause, or evolved to diapause as adults, either in a state of developmental quiescence or in reproductive diapause, which is characterized by state of arrested development of reproductive tissues (Santos et al., 2019). Potentially, the loss of PTTH and Torso from the social bee lineages included in our analysis (all of whom have evolved to diapause as adults, rather than earlier developmental stage) is evolutionarily connected to this reorganization of the timing of diapause.

## Discussion

Here, we demonstrate that a number of social bee lineages have lost a canonical regulator of moulting and metamorphosis, PTTH, and its receptor, Torso, and propose that this loss might be connected to an increasing reliance on social rather than abiotic cues in the developmental timing of offspring in social bee nests. Importantly, our inferences about the loss of PTTH within bees, and our hypothesis that this is connected with social evolution, are based on currently-available data and may change as more bee genomes are available for analysis. Given the relatively limited number of species included in our analysis (*n* = 22) and their phylogenetic distribution (e.g., relatively intensive sampling within the genera *Bombus* and *Apis*, both within the subfamily Apinae, and relatively low coverage of other groups), sequencing of additional genomes is required to shed further light on whether this pattern is observed more broadly across other bee families, and how extensive losses are within families included in our analysis. Interestingly, Skelly et al. (2019) found that PTTH has also been lost from some other insect groups, including *Blattella germanica*, eusocial termites (e.g., *Zootermopsis nevadensis*), and parasitic lineages in ants (e.g., *Pogonomyrmex barbatus*). Thus losses of this system have been detected in some other eusocial and parasitic lineages, but it is also retained in some eusocial lineages.

Inclusion of additional genomes into our analysis may alter our understanding of evolutionary patterns of PTTH and Torso in bees, and may change our interpretation of its association with sociality in bees. Further, only one subsocial bee was included in our analysis (*Ceratina calcarata*), and data from additional subsocial species are critical for identifying whether this lifestyle might also be broadly associated with the loss of PTTH and Torso. *Ceratina calcarata* exhibits prolonged maternal care and extensive parent-offspring interactions (Rehan and Richards, 2010). However, they live in cavities (twig nests) where temperature and other conditions within their nests are likely less regulated, given that their societies are typically very small (~2 individuals) relative to the larger, more controlled nests of eusocial lineages. A more complete picture of genome evolution in solitary and social bees, across all families, is necessary to fully understand how developmental processes have evolved within this insect group, in relation to social evolution or other evolutionary transitions.

## Data Availability Statement

Publicly available datasets were analyzed in this study. This data can be found at: http://HymenopteraGenome.org; https://www.ncbi.nlm.nih.gov/search/all/?term=PRJNA528016.

## Author Contributions

CC led the analyses, project, and authorship of the manuscript. NO contributed to the analyses and co-authored the manuscript. MO and NY contributed to the experimental design and co-authored the manuscript. SH co-led and co-designed the project and co-authored the manuscript. All authors contributed to the article and approved the submitted version.

## Conflict of Interest

The authors declare that the research was conducted in the absence of any commercial or financial relationships that could be construed as a potential conflict of interest.

## Publisher’s Note

All claims expressed in this article are solely those of the authors and do not necessarily represent those of their affiliated organizations, or those of the publisher, the editors and the reviewers. Any product that may be evaluated in this article, or claim that may be made by its manufacturer, is not guaranteed or endorsed by the publisher.

## Supplementary Material

The Supplementary Material for this article can be found online at: https://www.frontiersin.org/articles/10.3389/fphys.2022.831928/full#supplementary-material
