# Supplementary material for: Convergent Loss of Prothoracicotropic Hormone, A Canonical Regulator of Development, in Social Bee Evolution
Source: Front Physiol. 2022 Feb 15;13:831928. doi: 10.3389/fphys.2022.831928 (PMC8887954; doi:10.3389/fphys.2022.831928)
Supplement: Supplementary file 1 [file Data_Sheet_1.docx]

**SUPPLEMENTARY INFORMATION FOR:**

Costa CP, Okamoto N, Orr M, Yamanaka N, Woodard SH. Convergent loss of PTTH, a canonical regulator of development, in social bee evolution.

**Table S1.** Gene sequences used to examine presence/absent of genes from torso-activation cassette (TAC) in social and solitary bees.

| **Genes** | ***Drosophila melanogaster*** | ***Nasonia vitripennis*** |
| --- | --- | --- |
| *prothoracicotropic hormone (Ptth)* | NP_001285550.1 | XP_031783445.1 |
| *Torso* | NP_001097212.2 | XP_008211645.2 |
| *Trunk* | NP_476767.2 | NA |

**Table S2.** Species according to degree of social complexity. *Eufriesea mexicana is communal, but its social biology is relatively unknown.*

| **Genes** | ***Group*** | ***Status*** |
| --- | --- | --- |
| *Apis mellifera* | Apidae | Eusocial |
| *Apis dorsata* | Apidae | Eusocial |
| *Apis florea* | Apidae | Eusocial |
| *Apis cerana* | Apidae | Eusocial |
| *Frieseomellita varia* | Apidae | Eusocial |
| *Melipona quadrifasciata* | Apidae | Eusocial |
| *Bombus impatiens* | Apidae | Eusocial |
| *Bombus ignites* | Apidae | Eusocial |
| *Bombus terrestris* | Apidae | Eusocial |
| *Bombus vosnesenkii* | Apidae | Eusocial |
| *Bombus bifarius* | Apidae | Eusocial |
| *Bombus vancouverensis* | Apidae | Eusocial |
| *Eufriesea mexicana** | Apidae | Communal |
| *Ceratina calcarata* | Apidae | Subsocial |
| *Habropoda laboriosa* | Apidae | Solitary |
| *Megachile rotundata* | Megachilidae | Solitary |
| *Osmia bicornis* | Megachilidae | Solitary |
| *Osmia lignaria* | Megachilidae | Solitary |
| *Lasioglossum albipes* | Halictidae | Facultative eusocial |
| *Megalopta genalis* | Halictidae | Facultative eusocial |
| *Nomia melanderi* | Halictidae | Solitary |
| *Dufourea novaeanglia* | Halictidae | Solitary |


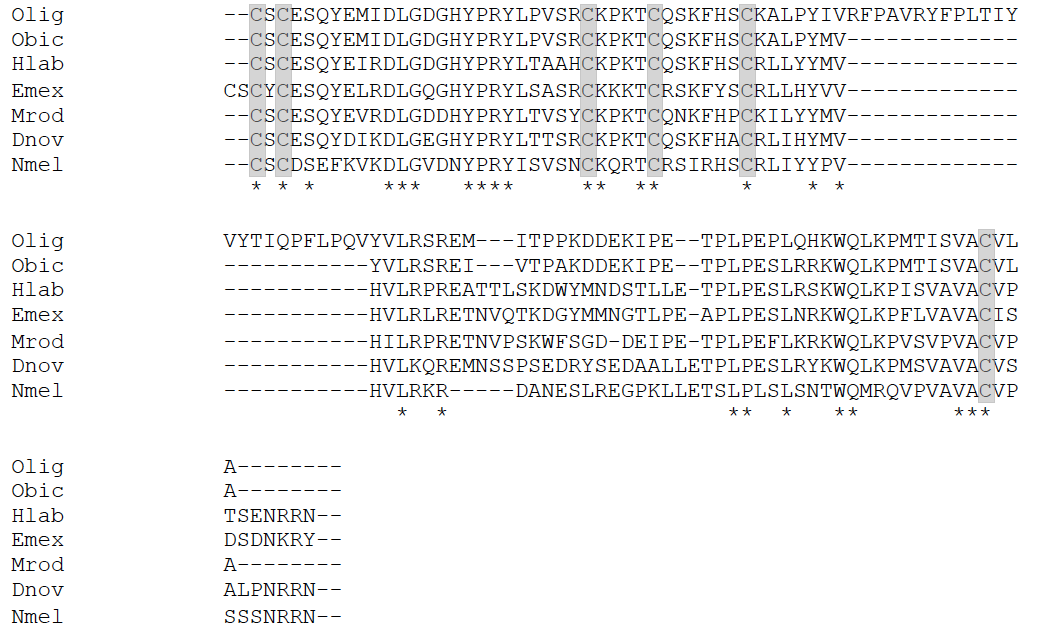


**Figure S1.** The alignment of partial PTTH amino acid sequences from bee species. Olig: *Osmia bicornis;* Obic: *Osmia bicornis;* Hlab: *Habropoda laboriosa;* Emex: *Eufriesea Mexicana;* Mrod: *Megachile rotundata;* Dnov: *Dufourea novaeanglia;* Nmel: *Nomia melanderi.* The conserved cysteine residues were highlighted in gray. **Alignment was partially modified manually to highlight the conserved cysteine residues.*
